# Potential Effects of Soccer Ball Characteristics on Ball-to-Head Contact: A Systematic Review

**DOI:** 10.3390/jfmk9040210

**Published:** 2024-10-27

**Authors:** José M. Oliva-Lozano, Carlos D. Gómez-Carmona, José M. Muyor, George T. Chiampas, Barry Pauwels, Rick Cost

**Affiliations:** 1 United States Soccer Federation, Chicago, IL 60601, USA; jlozano@ussoccer.org (J.M.O.-L.); gchiampas@ussoccer.org (G.T.C.); bpauwels@ussoccer.org (B.P.); rcost@ussoccer.org (R.C.); 2 Training Optimization and Sports Performance Research Group (GOERD), University of Extremadura, 10003 Caceres, Spain; 3 BioVetMed & SportSci Research Group, University of Murcia, 30100 Murcia, Spain; 4 Department of Music, Plastic and Body Expression, Human and Social Sciences Faculty, University of Zaragoza, 44003 Teruel, Spain; 5 Health Research Centre, Faculty of Educational Sciences, University of Almería, 04120 Almería, Spain; josemuyor@ual.es

**Keywords:** football, header, ball properties, biomechanics, concussion

## Abstract

**Background:** The aim of this study was to systematically review research on the effect of soccer ball characteristics on ball-to-head contact. **Methods**: This systematic review was conducted using electronic databases, which included PubMed, Cochrane, and Web of Science. The search strategy combined keywords related to soccer, the ball and its characteristics, heading, and kinematics variables. Studies analyzing the impact of soccer ball characteristics on heading biomechanics were included. The review included studies using mathematical models, simulations, and human subjects. **Results**: A total of nine studies were included, highlighting the lack of evidence on this topic. The following ball characteristics were investigated: inflation pressure (*n* = 7), mass (*n* = 4), structure/material properties (*n* = 3), size/diameter (*n* = 3), and stiffness (*n* = 3). Most studies used non-human subjects, such as mathematical, simulated, or head-form models. Key findings were as follows: (a) reducing inflation pressure may decrease impact magnitude; (b) ball size may not directly relate to impact magnitude, but one study found that a smaller size resulted in a shorter impact time; (c) lower impact observed with decreasing ball mass; (d) lowering stiffness showed a tendency to lower impact; (e) two studies on water absorption found that wet balls were heavier and had greater impact forces than dry balls; and (f) ball structure and cover material directly influenced impulsive forces. **Conclusions**: Modifying soccer ball characteristics may reduce heading forces, but the available research has limitations. More controlled studies are needed to determine optimal ball properties for mitigating injury risk during soccer heading. Standardized testing methods can further clarify the biomechanics of heading, supporting ongoing innovations to enhance player experience.

## 1. Introduction

Soccer is arguably the world’s most popular sport and it is a health-promoting activity for participants across the lifespan [1]. Soccer is considered an attractive way of enhancing aerobic fitness and preventing and treating non-communicable diseases (e.g., hypertension and type 2 diabetes), and it is ideal for addressing a lack of motivation [2]. For instance, playing soccer produces large improvements in maximum oxygen uptake (VO_2_ max) compared to strength training and no exercise, regardless of the age, sex, and health status of the participants [2]. Specifically, VO_2_ max increases by ~3.5 mL/kg/min during a recreational soccer training program in comparison with other types of training [2]. In addition, research has shown that youth soccer players were more often enrolled in the pre-university academic system (i.e., high academic achievers) compared to control groups of typical students [3].

Although, in soccer, the ball is played mainly with the foot, it can also be controlled, cleared, and hit with other parts of the body (except the hand/arm, which only goalkeepers can use). In this regard, purposeful heading is a unique part of soccer, allowing players to use their heads to advance the ball in play [4]. Youth players typically learn this skill once they have gone through the fundamentals of passing, dribbling, and shooting [4]. Youth soccer players head the ball at a low frequency, although this usually increases with age [4]. These repetitive head impacts are typically “low” magnitude impacts compared to the theoretical threshold of concussion (e.g., ~100 g) [4], but, clinically, decreases in peak linear and angular acceleration, as well as head impact power, may reduce the risk of acute and cumulative head injury [5]. Studies have shed light on the prevalence and nature of these injuries across various levels of play. In National Collegiate Athletic Association (NCAA) women’s soccer, concussions accounted for 11.4% of all injuries, compared to 7.0% in men’s soccer [6]. Another study reported that at the collegiate level, 6.2% of soccer players experienced a concussion each year [7]. A Canadian study analyzing soccer-related head injuries in youth (ages 5–29 years) found that 27.0% of these injuries were associated with heading the ball [7]. Notably, 12.4% of all soccer-related head injuries were diagnosed as concussions or other brain injuries, with concussions representing 40.15% of these injuries [8]. Although most impacts may not reach concussion levels, understanding the potential effects of repetitive low-magnitude impacts is important. These findings emphasize the opportunity to explore strategies that can reduce the risk of head injuries in soccer, such as improving the design and selection of equipment like soccer balls. Encouragingly, research continues to inform ways to keep the game safer while allowing players to enjoy the sport with confidence.

According to a recent review, playing position seems to influence the number of heading actions but has less influence on the magnitude of the acceleration of the head [9]. Thus, although heading biomechanics research in soccer is limited, measuring head accelerations during purposeful heading in soccer players has received special attention from a medical research perspective. In addition, a recent study suggested investigating adjustments to ball properties in order to mitigate heading burden in players across all levels of soccer [10]. Currently, the International Football Association Board (IFAB) Laws of the Game state that all balls must be spherical and made of suitable material, with the following characteristics: circumference (68–70 cm), weight (410–450 g), and pressure (0.6–1.1 atmosphere at sea level, 8.5–15.6 lbs/sq in) [11]. These are the characteristics of a size 5 ball; however, the rules and characteristics of the ball can vary with age group [12].

The Fédération Internationale de Football Association (FIFA) Football Quality Program has conducted research into the market and provided recommendations for different ball sizes and masses to be used in youth soccer for different age groups [13]. The impact of ball characteristics on heading may vary significantly across different age groups, making it crucial to consider age-specific modifications to enhance safety [13,14]. For instance, if there are differences in neck strength, heading technique, or experience, these could also play a role in minimizing accelerations [14].

In this regard, several studies showed that various features of the ball, such as the size, weight, ball pressure, or rate of absorbing water in wet conditions, may have an impact on head accelerations [15,16]. For example, it was observed that linear head accelerations decreased up to 59% when using a size 4 ball or the lightest version of a size 5 ball, compared to the officially regulated size 5 [16]. In response to growing interest in understanding and optimizing player safety, proactive approaches to enhance heading safety are of great relevance to soccer-related governing bodies [10].

As mentioned above, one of the approaches might be being proactive and considering modifications of the ball characteristics based on the age group, or even possibly gender. Age-specific and gender-specific considerations are particularly important, as the biomechanics of heading may vary considerably among children, adolescents, and adults, as well as between males and females [14,17]. However, a comprehensive review of the literature is necessary in order to have an evidence-based approach. Therefore, this study aims to systematically review the literature on the effect of the characteristics of the ball on ball-to-head contact, measured in humans or simulated environments or based on theoretical models.

## 2. Materials and Methods

### 2.1. Search Strategy

This systematic review was reported and developed following the Preferred Reporting Items for Systematic Reviews and Meta-Analysis (PRISMA) guidelines [18]. The protocol for this systematic review was registered on PROSPERO (“CRD42023471584”) and is available in full at the National Institute for Health Research.

This systematic review was conducted using electronic databases, which included PubMed, Cochrane, and Web of Science. The review included publications before 1 September 2024. The following search strategy by title and abstract was designed:

[“ball”] AND [“characteristics” OR “properties” OR “features” OR “mass” OR “weight” OR “circumference” OR “size” OR “diameter” OR “pressure” OR “force” OR “water absorption” OR “softness” OR “mass” OR “inflation”] AND [“soccer” OR “football”] AND [“head” OR “heading” OR “header” OR “ball-to-head”] AND [“acceleration” OR “kinematics” OR “impact” OR “force”] AND [“soccer” OR “football”].

### 2.2. Study Selection

Only studies meeting the following inclusion criteria were selected: (a) studies analyzing the potential effect of one or different ball types or one or different ball characteristics on heading; (b) the full text was available in English; and (c) included cohort studies, randomized controlled trials, and cross-sectional study design. The studies selected did not discriminate among age groups, sex, or skill levels. Also, patents, books, book chapters, and conference abstracts were not included in this review.

Two independent reviewers (J.M.O.-L. and C.D.G.-C.) selected the studies based on the inclusion and exclusion criteria. All references were stored in the Mendeley reference management system (Elsevier, Amsterdam, The Netherlands). Since duplicates were observed, these were removed. Then, the titles and abstracts were examined. Finally, articles were read in full text, and only the studies that met the inclusion criteria were included in this study. If there was any disagreement between the reviewers, a third reviewer (J.M.M.) was involved in the decision-making process. This third reviewer independently assessed the study in question and provided their opinion. The final decision was then made based on a majority consensus among all three reviewers. 

**Figure 1:**
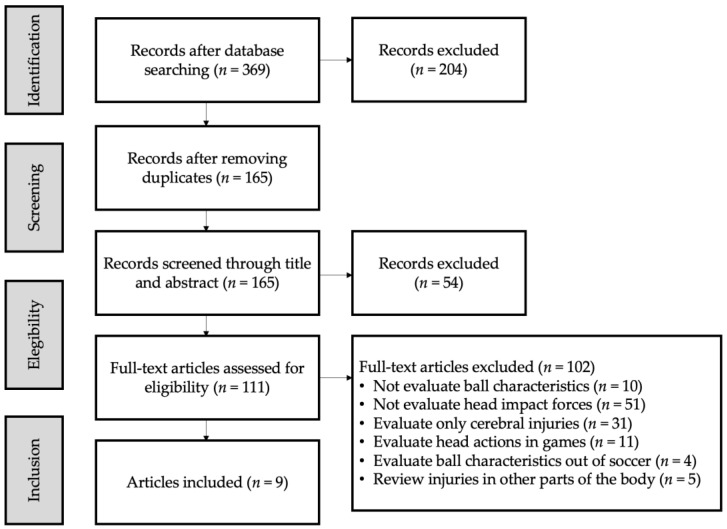
Characteristics of the selected studies for this systematic review.

A graphical description of the selection process may be observed in Figure 1. The main reasons for exclusion were as follows: (a) studies that did not evaluate head impact forces (*n* = 51); (b) studies that evaluated only cerebral injuries (*n* = 31); (c) studies that evaluated head actions in games without considering ball characteristics (*n* = 11); (d) studies that did not evaluate ball characteristics (*n* = 10); (e) studies that reviewed injuries in other parts of the body (*n* = 5); and (f) studies that evaluated ball characteristics outside of a soccer context (*n* = 4).

### 2.3. Data Abstraction

The following data were extracted from each study: author/s, year of publication, summary of the study protocol, description of the ball, ball characteristic being analyzed, outcome variable (i.e., kinematic variable), and main findings.

## 3. Results

### 3.1. Identification and Selection of Studies

A total of 369 studies were identified following the search strategy. Duplicates were removed, and a total of 204 titles and abstracts were examined. Of these, 111 were selected for full-text screening. Since 102 studies did not meet the inclusion criteria, 9 studies were selected for this study (Figure 1).

### 3.2. Characteristics of the Selected Studies

The characteristics of the selected studies are summarized in Table 1. The results of this review showed that the first study on this research topic was published in 2001 [19], and most studies available were conducted in the United States of America [12,19,20,21,22]. Specifically, Table 1 shows that the effect of the following characteristics of the ball on ball-to-head contact have been investigated: ball inflation pressure (*n* = 7) [12,15,16,19,20,21,22], ball mass (*n* = 4) [12,15,16,23], ball structure and specific material properties (*n* = 3) [12,15,24], ball size/diameter (*n* = 3) [12,16,21], and ball stiffness (*n* = 3) [15,21,23].

Another characteristic of the included studies was that most studies were based on non-human subjects, such as mathematical, simulated, or head-form models [12,19,20,21,22,23,24]. Only two studies included human subjects to measure the effects of different ball characteristics on impact kinematics-related variables [15,16].

### 3.3. Main Findings Related to Ball Inflation Pressure

This review found that reducing inflation pressure may lower the magnitude of impact. One study showed that the mean peak impact force for different inflation pressures (4 psi, 8 psi, 12 psi, and 16 psi) decreased with lower inflation pressures [12]. Babbs et al. [12] also observed a greater mean acceleration for a 1.1 atm ball (i.e., 16.2 psi) than the 0.6 atm (8.8 psi) and 0.3 atm (4.4 psi) balls, both in adult and youth soccer players [19]. In addition, the study found that brain accelerations during normal heading by adult players average less than 0.1% of accepted traumatic levels for a single impact [19]. Similar findings were obtained by others, in which the inflation pressure had a significant effect on linear acceleration [15,16,20,22] and rotational accelerations [15,16,22]. Specifically, one of the first studies published on this topic found that maximum linear acceleration, angular acceleration, total power, neck shear, and neck axial loads were lower as ball inflation pressure decreased (1.1, 0.8, 0.6, and 0.4 bar ≈ 15.9, 11.6, 8.7, and 5.8 psi, respectively) [15]. Finally, one of the studies that analyzed three different pressures based on a theoretical model (10, 12, and 14 psi) predicted an increase in the impact force of 7.98 N for an increase in the inflation pressure of the ball from 10 to 12 psi, but these differences were not considered substantial [21].

### 3.4. Main Findings Related to Ball Size/Diameter

Only three studies analyzed the effect of ball size/diameter [12,16,21]. Regarding the reviewed studies, it has been observed that ball size/diameter may not be directly related to the magnitude of the impact of the ball, but one study found that the smaller the size, the shorter the impact time [21]. Specifically, one of the studies concluded that altering the ball size was found to cause only minor differences in peak impact force and linear and angular head accelerations [21]. However, the ball size (e.g., size 3 vs. size 4 vs. size 5) was found to cause a substantial difference in the contact time between the ball and the head (i.e., the smaller the size, the shorter the impact) [21]. Another study with sizes 4, 4.5, and 5 observed that the size 4.5 ball produced the lowest impact [12]. In addition, the regression analyses from a different study did not show ball size to be a significant predictor of linear head acceleration (*p* = 0.45) and angular head velocity (*p* = 0.20) [16].

### 3.5. Main Findings Related to Ball Mass

Overall, this review found that a lower impact of the ball was observed with decreasing ball mass. For example, the EIR lightweight ball (0.381 kg) generated a mean impact force of ~2795 N, while the Adidas Starlancer size 4 (0.389 kg) and size 5 (0.432 kg) generated a mean of ~3044 N and ~3113 N [12]. In this regard, the regression analyses from a different study showed that ball mass was a significant predictor of linear acceleration (*p* < 0.001) and angular velocity (*p* < 0.001) (e.g., the lower the mass, the lower acceleration and velocity) [16]. In addition, peak linear accelerations in a different study decreased by 9–10% with a decreased ball mass of 20–32%, and head angular acceleration and neck shear/axial loads showed similar trends [15]. Moreover, another study found that the mathematical dynamic model (MADYMO) simulation mode, which was used for simulating contact forces during the soccer header reconstructions, illustrates that the force experienced by the head is directly proportional to the square root of the mass of the ball [23].

### 3.6. Main Findings Related to Ball Structure and Specific Material Properties

When it comes to analyzing kinematics through different ball structures and specific material properties, this review found two studies that looked at the water absorption of the ball [12,15] and one study that analyzed soccer balls with different surface materials and numbers of panels [24]. Although only two studies were found that were related to the water absorption of the ball, these studies observed that wet balls were heavier and generated greater impact forces than dry balls [12,15]. Regarding the structure of the ball and surface material, one of the studies observed that the structure of the ball and the flexibility of the surface material and the inner reinforcing layer could reduce the maximum impulsive forces [24]. 

### 3.7. Main Findings Related to Ball Stiffness

Although only three studies were found [15,21,23], a tendency toward lower impact with lower stiffness was observed. For example, the Mitre ball (25.9 kN/m) showed lower maximum linear acceleration, angular acceleration, total power, neck shear, and axial compression compared to the rest of the balls (Adidas World Cup 1974: 30.2 kN/m; Fevernova Tri-Lance: 33.6 kN/m; Adidas Santiago: 36.7 kN/m). In addition, the mathematical and MADYMO simulation model illustrated that the force experienced by the head was directly proportional to the square root of the stiffness of the ball, indicating that increasing ball stiffness results in a non-linear increase in head impact force [23]. However, a different study concluded that peak impact force, linear and angular head acceleration, and contact time were not found to be substantially altered by the increase in ball stiffness that occurred with increased inflation pressure [21].

The information about all studies that investigated the relationship between ball characteristics and head acceleration/forces is included in Table 2.

## 4. Discussion

The aim of this study was to conduct a systematic review of the literature about the effect of soccer ball characteristics on impact kinematics. A total of nine studies were included in this study, which shows the scarcity of evidence related to this topic. Specifically, the following characteristics of the ball have been investigated: inflation pressure, mass, structure, specific material properties, size/diameter, and stiffness. The main finding from this study, which is the first systematic review on this research topic, was that modifications to ball properties may reduce the magnitude of impact when heading the ball.

Research on this topic is scarce, which can be observed by the fact that only nine studies were included in this systematic review. Also, caution should be taken when interpreting the results of this review not only because of the lack of robust research but also because of the heterogeneity in the methodologies of the studies. Some studies were based on non-human subjects, such as mathematical, simulated, or head-form models [12,19,20,21,22,23,24], while others included human subjects [15,16], which limits the ability to compare between studies or conduct a meta-analysis. This heterogeneity, noted by previous researchers [10], highlights the need for more studies and high-quality research in this field. However, this is not a new research question since the first study was published in 2001 [19]. Specifically, this seems to have been an important topic in the United States of America [12,19,20,21,22], perhaps due to the high risk of concussion observed in other collision sports (e.g., athletes participating in sports like rugby, ice hockey, or American football have the highest incidence rates) [25].

Despite the lack of solid conclusions and the number of studies in this area, the main finding of this review is that adjustments to ball properties may be made to decrease the magnitude of impact when heading the ball. In this regard, a recent study concluded that the force experienced by the head during heading seemed to be mainly influenced by the speed of the ball [23]. However, ball speed is not necessarily controllable during gameplay, and limiting ball velocity in the game (e.g., in a free kick) may be an impractical adjustment [12]. On the other hand, reducing ball inflation pressure or ball mass may decrease the magnitude of ball-to-head impacts, and it is a practical adjustment. Also, although ball size/diameter may not be directly related to the magnitude of the impact of the ball, one study showed that the smaller the size, the shorter the impact time [21]. These are considerations that the governing bodies of soccer may implement as part of the rules of the game or adapt to different age groups.

In addition, soccer ball manufacturers, which play an important role in the design and construction of balls, should take into account other properties of the ball, like the stiffness, water absorption, structure, and type of materials that cover the ball. The reason is that a tendency to lower impact when lowering stiffness was observed [15,21,23], but it is important to note a main limitation from the last citation since a stationary head with maximal neck strength was simulated, and this is unrealistic in soccer [23]. However, the characteristic of ball stiffness (like the ball mass) is influenced by the pressure and material properties of the ball [23]. Also, wet balls absorb water, increasing their mass and, consequently, generate greater impact forces than dry balls [12,15]. This moisture-induced weight gain emphasizes the importance of ball mass as a key factor in impact force [12]. Furthermore, the structure of the ball and the type of materials that cover the ball may directly influence the impulsive forces [24]. For example, the maximum impulsive force for Cafusa was lower than that of the rest of the balls. However, the impulse for Jabulani was greater than that of the other balls [24]. When it comes to the Cafusa ball, strips of cloth are attached to fabricate the woven portion of the middle layer [24]. However, this portion in Jabulani is fabricated by using machine stitching, which has the characteristic of being stiffer than the cotton adhesion used for the middle-layer structure of Cafusa [24]. This characteristic may be one reason that a large impulse occurs when a large impulsive force is applied to Jabulani. Therefore, considering that official soccer balls undergo rigorous testing, as established by the FIFA Quality Program for footballs [26], these specific material properties could be considered to reduce the impact of heading the ball.

Although this review presents key findings, there are some major limitations that need to be acknowledged. The number of studies examining the effect of each specific ball characteristic on ball-to-head contact was very small, severely limiting the strength of conclusions that could be drawn. There was also a notable lack of standardized protocols and methods for data collection, which limits comparison between studies. Most included studies were based on mathematical models, simulations, or head-form models rather than human subjects, potentially limiting the ecological validity of findings. Additionally, a methodological quality assessment was not completed given the heterogeneity of the studies. Thus, this study does not fully answer other questions about heading in soccer, such as long-term neurological effects or differences across player demographics, including sex, age groups, or skill levels, among others.

Despite these limitations, this systematic review represents an important step in synthesizing the available evidence on the effects of soccer ball characteristics on heading dynamics. It provides valuable insights into how factors such as ball inflation pressure, mass, size, and material properties may influence impact forces during heading. The inclusion of both experimental and theoretical studies offers a comprehensive overview of current knowledge in this area. Furthermore, this review highlights specific areas in which further research is needed, potentially guiding future studies to address existing gaps in the literature. By consolidating findings from multiple studies, this review offers a foundation for developing evidence-based recommendations to enhance player safety in soccer, particularly regarding heading practices and equipment design.

## 5. Conclusions

Based on the results from this review, there are some potential practical applications to the game from a safety perspective. For example, using balls with lower inflation pressure and mass could help reduce the magnitude of ball-to-head contact. This is especially important when heading the ball at the youth level, in which the FIFA Youth Football Specification Recommendations for U9 (size 3: 600–620 mm and 280–310 g; or size 4 light: 635–660 mm and 290–320 g), U11 (size 4 light: 635–660 mm and 290–320 g; or size 4: 635–660 mm and 350–390 g), U13 (size 4: 635–660 mm and 350–390 g; or size 5 light: 680–700 mm and 350–380 g), and over 13 (size 5: 680–700 mm and 410–450 g) are the most updated guidelines available to date [13]. This implies that using the recommended ball mass, pressure, and size is important for each age group, especially in situations in which the coach/club does not provide players with balls because the players are the ones bringing balls to the session. In this specific case, it may be suggested that parents or legal guardians are provided with a list of standardized/recommended balls and specific characteristics.

In addition, the regular use of ball pressure gauges is highly recommended as an inflation pressure monitoring tool that ensures that balls conform to the ball manufacturer’s recommendations and specific age group guidelines. Also, if the official size 5 ball is used as established by the International Football Association Board (IFAB) Laws of the Game (weight: 410–450 g; pressure: 0.6–1.1 atmosphere at sea level, 8.5–15.6 lbs/sq) [11], the lowest ranges may be recommended for lower heading impact or specific heading drills or moments.

Also, considering that the specific material properties of the ball and its structure may have an influence on the ball-to-head contact, the use of certified balls is recommended. This is especially important in youth and amateur soccer, in which the quality of the balls may be lower compared to those used at the professional level. In addition, given wet environmental conditions, soccer balls may be cycled out of gameplay for new, dry balls [12]. This is due to the fact that balls with lower water resistance may be above the recommended weight thresholds under these conditions.

Finally, adding inertial sensors to the ball could provide valuable insights into how different ball properties affect ball-to-head contact. These sensors could provide precise data on ball acceleration, rotation, and impact force during heading. For example, they could measure peak linear and angular accelerations, impact duration, and force distribution across the ball’s surface. This technology could help quantify how changes in ball inflation pressure, mass, or material composition affect the biomechanics of heading. Future research using these sensors could offer insights into optimizing ball design for player safety while maintaining performance. From a practical perspective, this data could inform evidence-based guidelines for ball manufacturing and usage across different player demographics and skill levels.

## Figures and Tables

**Table 1:** Characteristics of the selected studies for this systematic review.

Authors (Year)	Study Design	Ball Characteristics	Dependent Variables
Auger et al. (2020) [12]	Two size 4 soccer balls, two size 5 balls, and two “lightweight” balls with different pressures were kicked by a researcher using a typical instep strike in sets of ten, ranging from low- to high-power kicks within each set, from a distance of 2 m far away from the force plate. A motion capture system with an integrated force plate was used to collect the data. Also, a submersion experiment was carried out to investigate the potential for mass increase during a 90 min match.	Inflation pressure Size/diameter Mass Ball structure and material properties	Mean peak impact force (N) Soccer ball mass (kg)
Babbs (2001) [19]	A set of simple mathematical models based on Newton’s second law of motion to describe the physics of heading was created.	Inflation pressure	Mean acceleration (g’s) Contact time (ms)
Cecchi et al. (2020) [22]	Size 5 soccer balls were launched by a ball launcher against an instrumented, anthropomorphic, test dummy head.	Inflation pressure	Linear acceleration (g) Rotational acceleration (krad/s^2^)
Koizumi et al. (2014) [24]	Size 5 soccer balls differing in surface material and number of panels were analyzed, and a kick-robot equipped with a dynamometer was used to measure the impulsive force at the time of impact.	Ball structure and material properties	Impulsive force (N/s)
Moscoso (2017) [20]	Size 5 soccer balls were launched by a ball launcher against an instrumented, anthropomorphic, test dummy head.	Inflation pressure	Peak linear impact acceleration (g) Angular velocity (deg/s)
Peek et al. (2021) [16]	Male and female soccer players (ages: 12–17 years) completed heading trials with four different balls while wearing a head-mounted accelerometer and gyroscope.	Inflation pressure Size/diameter Mass	Linear acceleration (g) Angular velocity (rad/s)
Queen et al. (2003) [21]	The following mathematical model inputs were obtained: elastic modulus and mass of size 3, size 4, and size 5 balls at inflation pressures of 10, 12, and 14 psi, head modulus, head mass, head length, head and trunk moment of inertia, and the precontact ball velocity. The model outputs consisted of linear and angular head acceleration, impact force, contact time between the ball and head, and head impact criteria.	Inflation pressure Size/diameter Stiffness	Linear acceleration (m/s^2^) Angular acceleration (rad/s^2^) Peak impact force (N) Contact time (ms)
Shewchenko et al. (2005) [15]	A validated numerical model and human subjects to quantify the biomechanical response of ball impact were used. Initial tests were conducted with human subjects to gain an understanding of heading techniques on biomechanical response and to validate the movements of a 50^th^-percentile male numerical model. Then, the numerical model was used to analyze the sensitivity of ball mass, pressure, construction, and condition on head response. The numerical model was exercised under a number of different ball mass and pressure properties and for a series of new and old generation balls (all size 5).	Inflation pressure Mass Ball structure and material properties Stiffness	Maximum linear acceleration (m/s^2^) Maximum angular acceleration (rad/s^2^) Maximum total power (kW) Maximum neck shear (N) Maximum neck axial (N)
Tierney et al. (2021) [23]	Forces experienced during heading using Mathematical DYnamic MOdels (MADYMO) for human body computational simulations.	Mass Stiffness	Force (N)

**Table 2:** Effect of ball features on heading-related variables.

Reference	Specific Ball Features	Results	Conclusions
	*Inflation pressure*		
Auger et al. (2020) [12]	Adidas Starlancer (sizes 4 and 5) and EIR Soccer lightweight ball (size 4.5) with the following different pressures: 0.27 bar, 4 psi 0.55 bar, 8 psi 0.83 bar, 12 psi 1.10 bar, 16 psi	Mean peak impact force for size 4, size 4.5 (lightweight ball), and size 5 0.27 bar, 4 psi: 2508 ± 368 N (size 4), 2440 ± 340 N (size 4.5, lightweight), 2669 ± 136 N (size 5) 0.55 bar, 8 psi: 2858 ± 455 N (size 4), 2688 ± 380 N (size 4.5, lightweight), 2895 ± 817 N (size 5) 0.83 bar, 12 psi: 3167 ± 444 N (size 4), 2961 ± 346 N (size 4.5, lightweight), 3284 ± 60 N (size 5) 1.10 bar, 16 psi: 3644 ± 334 N (size 4), 3093 ± 326 N (size 4.5, lightweight), 3606 ± 340 N (size 5)	A lower inflation pressure produces less ball impact, independently of the ball size.
Babbs (2001) [19]	Size 5 ball (mathematic model) 1.1 atm 0.6 atm 0.3 atm	The range of mean acceleration (g’s) for a 1.1 atm ball was greater than that of the 0.6 atm and 0.3 atm balls, both in adult and youth players. All ball impacts were considered as not damaging, with the highest accelerations associated with high ball velocity and pressure. Measures of touch increase as inflation pressure decreases.	It was observed that a lower inflation pressure produces less ball impact in adult and youth players with size 5 and size 4 balls, respectively.
Cecchi et al. (2020) [22]	Size 5 ball (Adidas) with the following different pressures: 76 kPa 62 kPa 55 kPa 48 kPa 34 kPa	Significant effects of pressure on peak linear acceleration (F = 72.86, *p* < 0.001, η^2^ = 0.960) and peak rotational acceleration (F = 4.81, *p* = 0.04). Balls inflated to 34 kPa resulted in lower peak linear acceleration than balls inflated to 55 kPa (p < 0.001), 62 kPa (*p* = 0.015), and 76 kPa (*p* < 0.001). Balls inflated to 76 kPa also produced greater peak linear acceleration than balls inflated to 55 kPa (*p* = 0.009). Significant reduction in peak rotational acceleration from 34 kPa balls relative to balls inflated to 76 kPa (*p* = 0.01).	A lower inflation pressure produces less ball impact.
Moscoso (2017) [20]	Size 5 ball (Adidas) with the following different pressures: 11 psi 9 psi 7 psi 5 psi	Significant effect of ball pressure on peak linear impact acceleration (F= 31.5, *p* < 0.001) but not on angular velocity (F= 1.35, *p* = 0.26). Overinflating the soccer ball to 76 kPa (11 psi) increased the linear impact acceleration by 7%, while underinflating to 34 kPa (5 psi) decreased this acceleration by 13.5%.	A lower inflation pressure produces less ball impact.
Peek et al. (2021) [16]	Four different balls (sizes 5 and 4) with the following pressures: 10.5 psi: Deploy Envision ball 5 psi: KickerBall, Adidas Starlancer ball, and Heading-Pro ball	Linear acceleration (min/max) and angular acceleration (min/max) KickerBall: 3.75 g (min)/9.88 g (max); 1.37 rad/s (min)/11.48 rad/s (max) Adidas Starlancer: 5.78 g (min)/16.51 g (max); 1.27 rad/s (min)/16.64 rad/s (max) Heading-Pro: 4.39 g (min)/12.34 g (max); 1.42 rad/s (min)/11.37 rad/s (max) Deploy Envision: 9.54 g (min)/21.51 g (max); 5.42 rad/s (min)/16.79 rad/s (max) Statistical differences between ball type and dependent variables (F=53.68, *p* <0.001). The regression analyses of predictors for linear acceleration and angular velocity found pressure to be a significant variable.	A lower inflation pressure produces less ball impact.
Queen et al. (2003) [21]	Three different pressures based on a theoretical model of 3 balls (sizes 3, 4, and 5): 10 psi 12 psi 14 psi	Inflation pressure does not substantially influence any of the impact characteristics during soccer heading The results of the present model predicted an increase in the impact force of 7.98 N for an increase in the inflation pressure of the ball from 10 to 12 psi, but these differences were not considered as substantial.	Inflation pressure does not have a substantial effect on impact variables. A tendency for lower inflation pressure to produce less impact is reported.
Shewchenko et al. (2005) [15]	Fevernova Tri-Lance ball (size 5) with the following different pressures: 1.1 bar 0.8 bar 0.6 bar 0.4 bar	Max. linear acceleration/Max. angular acceleration/Max. total power/Max. neck shear/Max. neck axial 1.1 bar: 170 m/s^2^/−402 rad/s^2^/1.53 kW/368 N/−574 N 0.8 bar: 156 m/s^2^/−374 rad/s^2^/1.44 kW/362 N/−570 N 0.6 bar: 150 m/s^2^/−363 rad/s^2^/1.38 kW/359 N/−566 N 0.4 bar: 107 m/s^2^/−286 rad/s^2^/1.04 kW/329 N/−539 N The head responses were reduced by up to 31%, with a ball pressure reduction of 50%. Reductions in the axial neck compression responses were noted for changes in ball pressure reductions of less than 25%, with much greater benefits for reductions above this percentage. Decreases in peak linear accelerations up to 10% were observed for a ball pressure decrease of 50%.	A lower inflation pressure produces less ball impact.
	*Size/Diameter*		
Auger et al. (2020) [12]	Adidas Starlancer ball Size 4: 0.205 m Size 5: 0.220 m EIR Soccer lightweight ball Size 4.5: 0.214 m	Mean peak impact force for size 4, size 4.5 (lightweight ball), and size 5 Size 4: 3044 ± 400 N Size 4.5: 2795 ± 348 N Size 5: 3113 ± 338 N Size 4.5 lightweight ball produces lower impact than size 4 and size 5 Adidas Starlancer.	Ball size/diameter is not directly related to the impact of the ball.
Peek et al. (2021) [16]	Four different balls: Size 5: KickerBall, Adidas Starlancer ball, and Deploy Envision ball Size 4: Heading-Pro	Linear acceleration (min/max) and angular acceleration (min/max) Size 5, KickerBall: 3.75 g (min)/9.88 g (max); 1.37 rad/s (min)/11.48 rad/s (max) Size 5, Adidas Starlancer: 5.78 g (min)/16.51 g (max); 1.27 rad/s (min)/16.64 rad/s (max) Size 5, Deploy Envision: 9.54 g (min)/21.51 g (max); 5.42 rad/s (min)/16.79 rad/s (max) Size 4, Heading-Pro: 4.39 g (min)/12.34 g (max); 1.42 rad/s (min)/11.37 rad/s (max) Statistical differences between ball type and dependent variables (F=53.68, *p* < 0.001). However, the regression analyses of predictors for linear acceleration and angular velocity did not show ball size to be a significant variable.	Ball size/diameter is not directly related to the impact of the ball.
Queen et al. (2003) [21]	Radius (m) Size 3: 0.09 Size 4: 0.10 Size 5: 0.11	As the size of the ball decreased, the contact time values decreased. However, altering the ball size was found to cause only minor differences in the peak impact force and the linear and angular head accelerations.	Ball size/diameter is not directly related to the impact of the ball.
	*Mass*		
Auger et al. (2020) [12]	Adidas Starlancer Size 4: 0.389 kg Size 5: 0.432 kg EIR Soccer lightweight Size 4.5: 0.381 kg	Mean peak impact force for size 4 ball, size 4.5 (lightweight ball), and size 5 ball Size 4: 3044 ± 400 N Size 4.5: 2795 ± 348 N Size 5: 3113 ± 338 N Size 4.5 lightweight ball produces lower impact than size 4 and size 5 Adidas Starlancer.	A lower impact of the ball was observed when decreasing the ball mass.
Peek et al. (2021) [16]	Size 5: KickerBall, 192 g Size 5: Adidas Starlancer, 432 g Size 4: Heading-Pro, 255 g Size 5: Deploy Envision, 430 g	Linear acceleration (min/max) and angular acceleration (min/max) KickerBall: 3.75 g (min)/9.88 g (max); 1.37 rad/s (min)/11.48 rad/s (max) Adidas Starlancer: 5.78 g (min)/16.51 g (max); 1.27 rad/s (min)/16.64 rad/s (max) Heading-Pro: 4.39 g (min)/12.34 g (max); 1.42 rad/s (min)/11.37 rad/s (max) Deploy Envision: 9.54 g (min)/21.51 g (max); 5.42 rad/s (min)/16.79 rad/s (max) Statistical differences between ball type and dependent variables (F = 53.68, *p* < 0.001). The regression analyses showed that ball mass was a significant predictor of linear acceleration (*p* < 0.001) and angular velocity (*p* < 0.001).	A lower impact of the ball was observed when decreasing the ball mass.
Shewchenko et al. (2005) [15]	Size 5 balls: Fevernova Tri-Lance: 0.44 kg Fevernova Junior 290: 0.30 kg Fevernova Junior 350: 0.35 kg Mitre: 0.43 kg Adidas WC 1974: 0.44 kg Adidas Santiago: 0.44 kg	Max. linear acceleration/Max. angular acceleration/Max. total power/Max. neck shear/Max. neck axial Fevernova Tri-Lance: 156 m/s^2^/−374 rad/s^2^ /1.44 kW/362 N/−570 N Fevernova Junior 290: 130 m/s^2^/−293 rad/s^2^/1.21 kW/279 N/−497 N Fevernova Junior 350: 140 m/s^2^/−330 rad/s^2^/1.30 kW/307 N/−532 N Mitre: 132 m/s^2^/−330 rad/s^2^/1.24 kW/341 N/−551 N Adidas World Cup 1974: 158 m/s^2^/−402 rad/s^2^/1.44 kW/369 N/−574 N Adidas Santiago: 164 m/s^2^/−390 rad/s^2^/1.49 kW/366 N/−573 N The peak linear accelerations decreased by 9–10% with a decreased ball mass of 20–32 %. The head angular acceleration and neck shear/axial loads showed similar trends, indicating that a lighter ball mass provides a net benefit for all measures of head/neck response	A lower impact of the ball was observed when decreasing the ball mass.
Tierney et al. (2021) [23]	Size 5 ball (simulated) 0.25–0.65 kg	Force (N) in mathematical/MADYMO simulation 0.25 kg: 950/850 N 0.65 kg: 1525/1400 N. The mathematical and MADYMO simulation model illustrates that the force experienced by the head is directly proportional to the square root of the mass of the ball.	A lower impact of the ball was observed when decreasing the ball mass.
	*Structure and material properties*		
Auger et al. (2020) [12]	Adidas Starlancer (sizes 4 and 5) EIR Soccer lightweight ball (Size 4.5)	Change in soccer ball mass (kg) during water submersion every 15′: 0′/15′/30′/45′/60′/75′/90′ Adidas Starlancer—Size 4: 0.39/0.47/0.46/0.46/0.48/0.47/0.48 kg EIR Soccer lightweight ball—Size 4.5: 0.38/0.40/0.42/0.42/0.42/0.42/0.42 kg Adidas Starlancer—Size 5: 0.43/0.56/0.59/0.58/0.59/0.59/0.58 kg The main change in weight is produced in the first 15 min. From 15 to 90 min, the weight changes are not significant.	Water absorption by these soccer balls may result in ball masses that substantially increase impact force. Specifically, the lightweight ball obtained lower water absorption in comparison to conventional size 4 and size 5 balls.
Koizumi et al. (2014) [24]	Size 5 ball Cafusa: 32 panels (6 spherical shapes × 4 panels + 8 outside panels) Jabulani: 8 triangular and tripod-shaped panels. TeamGeist2: 14 panels (6 propeller panels and 8 rotor panels). Pelias: 32 panels (12 pentagonal panels and 20 hexagonal panels)	Impulse force at 25 m/s Jabulani: 12.11 N*s Pelias: 11.89 N*s TeamGeist2: 11.87 N*s Cafusa: 11.84 N*s Only differences in force (N*s) were found at 25 m/s (not at 15, 20, and 30 m/s), at which Jabulani obtained a higher impact than the others (*p* < 0.05).	The maximum impulsive force for Cafusa was lower compared to the rest of the balls. This may be due to the inner reinforcing layer and flexibility of the surface material. The impulse for Jabulani was greater than that of the other balls at all velocities, which may be because of the characteristics of the structure of Jabulani and the surface material.
Shewchenko et al. (2005) [15]	Size 5 balls Weight dry/wet in kg (% diff.) Fevernova Tri-Lance: 0.44/0.46 (3%) Mitre: 0.43/0.60 (41%) Adidas WC 1974: 0.44/0.53 (22%) Adidas Santiago: 0.44/0.46 (4%)	Dry vs. wet (Max. linear acceleration/Max. angular acceleration/Max. total power/Max. neck shear/Max. neck axial) Fevernova Tri-Lance: 156 vs. 173 m/s^2^/−374 vs. −410 rad/s^2^/1.44 vs. 1.55 kW/362 vs. 376 N/−570 vs. −578 N Mitre: 132 vs. 174 m/s^2^/−330 vs. −446 rad/s^2^/1.24 vs. 1.55 kW/341 vs. 433 N/−551 vs. −608 N Adidas World Cup 1974: 158 vs. 180 m/s^2^/−402 vs. −419 rad/s^2^/1.44 vs. 1.58 kW/369 vs. 398 N/−574 vs. −582 N Adidas Santiago: 164 vs. 170 m/s^2^/−390 vs. −404 rad/s^2^/1.49 vs. 1.53kW/366 vs. 376N/−573 vs. −579N The percentage of mass increased due to the fact that water absorption varied considerably and exceeded the FIFA Quality specifications in some cases.	The neck and head responses increased in the wet condition due to the water uptake. The percentage of mass increase due to the fact that water absorption varied considerably.
	*Stiffness*		
Queen et al. (2003) [21]	10 psi: 22.1 N/mm (size 3)/22.9 N/mm (size 4)/22.2 N/mm (size 5) 12 psi: 24.9 N/mm (size 3)/25.5 N/mm (size 4)/24.9 N/mm (size 5) 14 psi: 26.7 N/mm (size 3)/28.1 N/mm (size 4)/28.0 N/mm (size 5)	Peak impact force, linear and angular head acceleration, and contact time were not found to be substantially altered by the increase in ball stiffness that occurred with increased inflation pressure.	Stiffness does not have a substantial effect on ball impact.
Shewchenko et al. (2005) [15]	Four balls with different stiffness: Mitre: 25.9 kN/m Adidas WC 1974: 30.2 kN/m Fevernova Tri-Lance: 33.6 kN/m Adidas Santiago: 36.7 kN/m	Max. linear acceleration/Max. angular acceleration/Max. total power/Max. neck shear/Max. neck axial compression Mitre: 132 m/s^2^/−330 rad/s^2^/1.24 kW/341 N/−551 N Adidas World Cup 1974: 158 m/s^2^/−402 rad/s^2^/1.44 kW/369 N/−574 N Fevernova Tri-Lance: 156 m/s^2^/−374 rad/s^2^/1.44 kW/362 N/−570 N Adidas Santiago: 164 m/s^2^/−390 rad/s^2^/1.49 kW/366 N/−573 N	A moderate increase in the values was found when increasing stiffness.
Tierney et al. (2021) [23]	Size 5 ball (simulated) 25–45 kN/m	Force (N) in mathematical/MADYMO simulation 25 kN/m: 1000/925 N 45 kN/m: 1350/1250 N The mathematical and MADYMO simulation model illustrates that the force experienced by the head is directly proportional to the square root of the stiffness of the ball.	A lower stiffness produces less ball impact.

## Data Availability

No new data were created or analyzed in this study.
